# Changes of children's physical activity from 1st to 4th grade are related to parents' educational level and Family Health Climate: a longitudinal study with primary school-aged children

**DOI:** 10.3389/fspor.2025.1537854

**Published:** 2025-06-09

**Authors:** Alexandra Ziegeldorf, Christina Niermann, Andreas Speer, Heike Streicher, Petra Wagner, Hagen Wulff

**Affiliations:** ^1^ Faculty of Sport Science, Leipzig University, Leipzig, Germany; ^2^ Institute of Interdisciplinary Exercise Science and Sports Medicine, Medical School Hamburg, Hamburg, Germany

**Keywords:** change pattern, physical activity, organized sport, health behavior, family, parent, children, Family Health Climate

## Abstract

**Introduction:**

Despite the well-known health benefits of physical activity, 81% of adolescents are physically inactive. Overall, studies showed that physical activity decreases with age during childhood and from childhood to adolescence. However, physical activity does not change uniformly for all children, the changes differ inter-individually. There are several studies that examine correlates and predictors of different patterns of change. However, studies focusing on family environmental factors are rare. The current study examines the relevance of two types of family environmental influences—parents' educational level and the Physical activity related Family Health Climate—for physical activity change patterns from first to fourth grade in primary school.

**Methods:**

Longitudinal data sets from the KOMPASS(2) study (*n* = 497) were used for analyses. Parents' educational level, Family Health Climate and children's overall physical activity as well as their engagement in organized sports were measured using parent questionnaires. Four groups were formed to map patterns of change for both children's overall physical activity and for their engagement in organized sports (1st to 4th grade): (1) no/not enough engagement at both time points, (2) a change from no/not enough engagement to (enough) engagement, (3) a change from (enough) engagement to no/not (enough) engagement and (4) (enough) engagement at both time points. Data were analyzed using multinomial logistic regression.

**Results:**

More than half of the children (53.5%) were in the pattern “continuously insufficient physical activity”, with a higher proportion of girls. For participation in organized sport, most children (56.1%) were assigned to the “continuous organized sport” pattern of change. Results showed differences in overall physical activity patterns according to parents' educational level and children's sex. Girls and children with highly educated mothers had an increased risk of being continuously physically inactive over the course of primary school. Organized sport patterns are related to parents' educational level and Family Health Climate. Children with higher educated parents and a higher Family Health Climate score had a lower risk of not being active in organized sport.

**Discussion:**

The study investigated characteristics of different patterns of physical activity change from 1st to 4th grade during primary school. It highlights the importance of differentiating between different types of physical activity as children's sex, parents' educational level and the Family Health Climate predicted overall physical activity and organized sports participation in different ways. This is the first study focusing on change patterns during primary school and stresses previous findings of an early decline of physical activity. The results indicate that the entry in the educational system might be a good time to start with interventions.

## 1 Introduction

Physical activity (PA) is beneficial for children's physical and mental health and prevents diseases such as obesity, diabetes, heart disease, metabolic syndrome or musculoskeletal disorders (1–5). However—despite decades of extensive efforts of research and practice—we still face the problem that too many youths (81%) do not reach the WHO recommendation of 60 min of moderate to vigorous PA per day (6), and therefore are not able to benefit from health enhancing effects of PA. There is still a need for studies generating data, that enhances the understanding of youths' health behavior.

Overall, studies showed that PA decreases with age and that PA levels in childhood predict PA levels in adulthood (7–10). However, it has been shown that PA does not develop uniformly, instead changes across lifespan are inter-individually different (11). Recent results identified various subgroups, indicating that studying PA trajectories or change patterns can yield new information on the complexity of PA behavior compared to studying population mean PA level only (11). The recognition of different PA change patterns and factors that are related to these patterns, is important for developing targeted PA promotion programs. Therefore, more in depth knowledge regarding correlates and predictors of change patterns is crucial (11). Additionally, it is important to differentiate PA domains (e.g., sports participation, overall PA) as correlates/predictors differ across PA types (12) and thus, patterns of change may also differ depending on the PA type and its associated correlates or predictors.

Studies focusing on PA change patterns showed that sociodemographic (e.g., educational level), behavioral (e.g., television viewing time) and family related influences (e.g., parental encouragement) are associated with PA changes over time (11, 13–15).

Associations between parents’ sociodemographic factors such as parents' educational level and children's PA have been extensively studied (16–20). However, the results are inconsistent. The correlations vary depending on the type of PA, e.g., organized vs. unorganized PA. For example, recent reviews found no association between parents' educational level and children's overall PA, but also indicated that children of higher educated parents, especially of higher educated mothers, engaged in less outdoor play and PA in general (20–22). However, there are other studies that found a positive relation between well-educated parents and habitual PA levels of their offspring while there was no correlation to children's engagement in organized sport (23). In contrast, other results indicate a positive correlation between parents' education and children's organized sport (24–26).

The family is the most important social context where healthy or unhealthy behavior patterns develop and are maintained (21). Theoretical frameworks in this context aim to describe a wide variety of family environmental factors and their interaction. For example, Frederick and Eccles (27) describe sociodemographic characteristics (e.g., education, family income, occupation) as a shaping factor for parents' beliefs and behaviors in general (e.g., personal values, parenting styles or sex stereotyped beliefs) and child-specific beliefs (e.g., perceptions of child's abilities and interests) which in turn affect child's motivation and behavior (27). The Levels of Interacting Family Environmental Subsystems (LIFES) organizes different family environmental influences and integrates influences arising from individuals, parent-child dyads and the family as a whole (28). It has been shown that beyond parents' individual factors (e.g., parents PA) and dyadic influences between parents and children (e.g., role modeling), there are family level socialization dynamics that affect children's health-related behaviors. In terms of the LIFES framework examples are family functioning (29–31) on a general and distal level, the Family Health Climate (FHC) (32) on a behavior-specific and proximal level, and joint family PA on a practical and immediate level.

Research on family influences on children's PA mostly focuses on dyadic influences (namely parent-child) such as the association between parenting behavior (e.g., parenting practices) and children's behavior. However, family is more than interactions between parents and children. According to families as systems approaches, the family is a complex interacting system and an organized whole (33). Family Systems Theory assumes that the elements of the system are interconnected and interdependent so that it could be best understood when viewed as a whole (33, 34). Therefore, variables that address this “family as a whole” thinking should be included in the efforts to explain children's PA (35). An important variable at the family-level is the FHC (32). The FHC reflects shared perceptions and cognitions concerning health and health behavior. It comprises individual experiences of daily family life, behavioral routines and interaction patterns within the family. Therefore, it serves as a framework for an individual's daily health behavior (32). Previous studies showed that PA related FHC (FHC_PA_) is associated with joint family PA and the amount of children's weekly PA (36).

The study focuses on different change patterns of children's organized and overall PA from 1st to 4th grade in primary school. The purpose is to (i) characterize the change patterns by means of children's sex, parents' educational level and families' Family Health Climate (FHC_PA_) and (ii) examine the predictive value of these variables in relation to the patterns of change. We assume that the distribution of children's sex and parents' educational level, and that the FHC_PA_ values differ across change patterns. Furthermore, we assume that these variables are predictors of group membership in terms of these change patterns.

## 2 Methods

### 2.1 Study design

Data for this analysis was obtained from the KOMPASS(2)-study. KOMPASS(2) is a survey that includes data from 30 primary schools in Leipzig, Germany. The study focuses on a longitudinal monitoring of primary school children's motor skills, anthropometric and clinical parameters (measured in grades one, two, and four) in combination with sociodemographic parameters, behaviors (e.g., PA, screen time) and behavioral determinants (e.g., social support, outcome expectations) (measured in grades one, three, and four; due to organizational constraints, no data could be collected in grade three). A total number of *N* = 1,140 primary school-aged children participated at baseline measurements in 2014/15 (first grade). Change patterns were calculated based on the first and the last measurement point (grade 1 and grade 4). Data from *n* = 497 children with complete data from both measurements were included.

### 2.2 Enrollment and participation

Prior to recruiting schools, the project team obtained the approval from the Saxonian State Office for School and Education. Afterwards, 65 elementary schools in Leipzig were invited to participate. A total number of 30 elementary schools gave their consent to participate. Subsequently, all parents or legal guardians of the participating children were informed about the study and the measurement procedures by written information and written informed consent was obtained. All information for participants was provided by the research team through the school. Ethical approval was obtained from the Ethics Committee of the Faculty of Medicine of the Leipzig University (Germany) in 2014 (File number: 253-14-14072014).

### 2.3 Measurements

Measurement procedures were identical for all measurement time points. Parents' educational level, FHC_PA_ and children's PA was assessed via a parent paper-pencil questionnaire. The questionnaires were sent to participating schools at each measurement point, were distributed by school teachers and filled out at home by parents. The completed questionnaires were collected by teachers and were picked up by the research team.

#### 2.3.1 Parents' educational level

Mothers' and fathers' educational level were assessed by asking for the highest school qualification. According to the German tripartite school system the categories ranged from “no qualification” to “university-entrance diploma.” Educational levels were cumulated to three categories: lower education (no school-leaving qualification and lowest German school qualification), intermediate education (intermediate German school qualification) and higher education (university-entrance diploma).

#### 2.3.2 Physical activity related Family Health Climate (FHC_PA_)

The FHC-Scale for physical activity (FHC_PA_) was used (32). The scale consists of three subscales with a total of 14 items. Subscales: value (e.g., “In our family it is normal to be physically active in our leisure time”), cohesion [e.g., “…we have fun doing physical activities together” (e.g., bike tours, hikes)] and information [e.g., “…we collect information (e.g., on the internet) on PA and exercise”]. The items were rated on a 4-point Likert-type scale (0 = “definitely false”, 1 = “rather false”, 2 = “rather true”, 3 = “definitely true”). A mean score of all FHC_PA_ items was calculated. The internal consistency in this study was *α* = .86.

#### 2.3.3 Children's PA and change patterns

The assessment of children's overall PA in grade 1 and grade 4 was inspired by Kohl and colleagues (37) and Schmidt and colleagues (38) asking how long the child usually is physically active during a day, with a medium to high intensity (indicator: sweating). Response options were: “not daily”, “up to 30 min. daily” (0–29 min), “up to 1 h daily”, “up to 2 h daily”, “over 2 h daily”. Following the current WHO recommendations (39) of at least 60 min of moderate to vigorous physical activity (MVPA) per day, answers were categorized in: “sufficient PA” if they reach the recommendations and “not sufficient PA” if not.

To map the changes of children's overall PA over time, *a priori* set groups were build: “continuously sufficient PA” when children reached the recommendations of at least 60 min of MVPA per day in grade 1 and 4, “decline” when children reach the recommendations in grade 1 but not in grade 4, “increase” when children did not reach the recommendations in grade 1 but in grade 4 and “continuously insufficient PA” when children have not reached the recommendations at both time points.

Regarding organized sports, parents were asked if their children regularly engage in sports in an organized setting (e.g., sports club) with the answer options “yes” or “no”. Following groups reflect the changes of the participation status from first to fourth grade: “continuously organized sport” when children participate in organized sports at the beginning and the end of primary school, “change from organized to no organized sport” when children participate in grade one but not in grade four, “change from no organized to organized sport” when children did not participate in grade one but in grade four and “no organized sport” when children did not participate in organized sports at both time points.

#### 2.3.4 Data analysis

Analyses were conducted with the longitudinally data set covering the measurements in the first and the fourth grade of primary school. The data was checked for plausibility and missing values were examined for systematic errors and subsequently replaced using the multiple imputation method (40). All statistical analyses were performed with SPSS Version 26.0 (IBM Corp., NY, USA). For analyzing group differences regarding the change patterns of organized and overall PA depending on sociodemographic factors and FHC one way Analyses of Variance (ANOVA), Pearson's chi-squared test and—in case of group differences with a cell frequency of less than five—the Fisher's Exact Test were performed. For analyzing the strength of association Cramer's *V* and Epsilon were used. Whereas Eta squared systematically overestimates the variance explained, Epsilon squared was used because this effect measure is less susceptible to bias (41).

In order to quantify the prediction of children's PA change patterns, multinomial logistic regression analyses were performed. To exclude multicollinearity, Pearson correlation was performed for the predictor variables. Using cut-off by Pituch & Stevens (42) there was no multicollinearity for this analysis (*r* < .7).

In the case of non-normally distributed data, bootstrapping procedure was used to obtain estimates. For calculating bias-corrected 95% confidence intervals, 1,000 bootstrapping iterations were requested.

## 3 Results

Table 1 shows the sample characteristics in grade 1 (baseline). Mean age of the children was 7.0 years (SD = .36), 51.7% were female. 57.1% of mothers and 49.1% of fathers have a high educational level. In total, 64.4% of the children engage in sports in organized settings such as sports clubs, with a higher percentage of boys. Regarding overall PA, 32.8% meet the WHO recommendations of at least 60 min of MVPA per day with a higher percentage of boys as well.

**Table 1:** Sample characteristics (*n* = 497).

Variable	Boys (*n* = 240)	Girls (*n* = 257)	Total (*n* = 497)
Age in years [M (SD)]	7.0 (0.4)	6.9 (0.4)	7.0 (0.4)
Maternal educational level			
Lower (%)	5.0	3.9	4.4
Intermediate (%)	37.9	38.9	38.4
Higher (%)	57.1	57.2	57.1
Paternal educational level			
Lower (%)	11.3	8.2	9.7
Intermediate (%)	37.1	45.1	41.2
Higher (%)	51.7	46.7	49.1
Organized physical activity			
Yes (%)	68.8	60.3	64.4
No (%)	31.3	39.7	35.6
Overall physical activity			
WHO recommendation achieved (%)	37.5	28.4	32.8
WHO recommendation not achieved (%)	62.5	71.6	67.2
Family Health Climate			
FHC_PA_ score [M (SD)]	1.81 (.50)	1.76 (.45)	1.78 (.48)

### 3.1 Characteristics of children's PA change patterns

The differences between the change patterns of overall PA are shown in Table 2. The majority of children (53.5%) were part of the change pattern “continuously insufficient PA”—they neither reached the recommendation of 60 min in PA per day at grade 1 nor at grade 4. Similar proportions (13.7 and 15.1%) were in the groups that increased their overall PA and decreased their overall PA from grade 1 to grade 4. Less than twenty percent (17.7%) were assigned to the change pattern “continuously sufficient PA”. The patterns “increase” and “decline” included a similar proportion of boys and girls while in the “continuously sufficient PA” pattern 62.5% of the children were boys and in the “continuously insufficient PA” pattern 56.4% were girls.

**Table 2:** Differences between the four change patterns of children's overall PA.

Predictors		Change patterns				Tests
		Continuously sufficient PA	Increase	Decline	Continuously insufficient PA	
Total [% (*n*)]		17.7 (88)	13.7 (68)	15.1 (75)	53.5 (266)	
Sex [% (*n*)]	Male	62.5 (55)	50.0 (34)	46.7 (35)	43.6 (116)	*χ*^2^ = 9.61 (3), *p* = .022, *V* = .022
	Female	37.5 (33)	50.0 (34)	53.3 (40)	56.4 (150)	
Maternal educational level [% (*n*)]	Lower	6.8 (6)	4.4 (3)	5.3 (4)	3.4 (9)	Fisher's exact test [*p* = (.026)], *V* = .035
	Intermediate	46.6 (41)	29.4 (20)	49.3 (37)	35.0 (93)	
	Higher	46.6 (41)	66.2 (45)	45.3 (34)	61.7 (164)	
Paternal educational level [% (*n*)]	Lower	14.8 (13)	4.4 (3)	13.3 (10)	8.3 (22)	Fisher's exact test [*p* = (.189)], *V* = .186
	Intermediate	40.9 (36)	36.8 (25)	44.0 (33)	41.7 (111)	
	Higher	44.3 (39)	58.8 (40)	42.7 (32)	50.0 (133)	
FHC_PA_ [M (SD)]		1.85 (.52)	1.78 (.44)	1.78 (.47)	1.76 (.47)	*F* = 0.85 (3, 493), *p* = .469, ${\hat{\varepsilon }}^{2}=-.00$

*V*, Cramer's *V*; ${\hat{\varepsilon }}^{2}$, Epsilon.

Interestingly, we found differences regarding maternal educational level depending on the change pattern: in the “increase” and the “continuously insufficient” group the proportion of highly educated mothers is the highest compared to the other educational levels. In contrast, the distribution in the group “continuously sufficient PA” of higher and intermediate maternal education levels is equal, and in the group “decline”, the proportion of mothers with an intermediate education level is highest. There were no differences regarding the educational levels of fathers. The change patterns of overall PA did not differ with regard to FHC_PA_ value.

For organized sports, more than half of the children were assigned to the change pattern “continuously organized sport” (56.1%), followed by “change from no organized to organized” (19.5%) and “no organized sport” (16.1%). The smallest proportion of children were part of the group “change from organized to no organized sport” (8.2%).

Regarding sex, more boys (53.4%) were in the group “continuously organized sport” while the proportion of girls in the other groups tended to be higher. The group “change from organized to no organized sport” showed the highest difference with more girls than boys quitting organized sports from 1st to 4th grade in primary school (61.0% vs. 39.0%) (Table 3).

**Table 3:** Differences between the four change patterns of children's engagement in organized sports.

Predictors		Change patterns				Tests
		Continuously organized sport	Change from no organized to organized sport	Change from organized to no organized sport	No organized sport	
Total [% (*n*)]		56.1 (279)	19.5 (97)	8.2 (41)	16.1 (80)	
Sex [% (*n*)]	Male	53.4 (149)	42.3 (41)	39.0 (16)	42.5 (34)	*χ*^2^ = 6.81 (3), *p* = .078, *V* = .078
	Female	46.6 (130)	57.7 (56)	61.0 (25)	57.5 (46)	
Maternal educational level [% (*n*)]	Lower	2.2 (6)	7.2 (7)	4.9 (2)	8.8 (7)	Fisher's exact test [*p* < (.001)], *V* < .001
	Intermediate	33.7 (94)	33.0 (32)	48.8 (20)	56.3 (45)	
	Higher	64.2 (179)	59.8 (58)	46.3 (19)	35.0 (28)	
Paternal educational level [% (*n*)]	Lower	7.5 (21)	12.4 (12)	9.8 (4)	13.8 (11)	Fisher's exact test [*p* < (.001)], *V* < .001
	Intermediate	35.1 (98)	41.2 (40)	46.3 (19)	60.0 (48)	
	Higher	57.3 (160)	46.4 (45)	43.9 (18)	26.3 (21)	
FHC_PA_ [M (SD)]		1.85 (.45)	1.78 (.50)	1.65 (.42)	1.63 (.52)	*F* = 5.63 (3, 493), *p* = .001, ${\hat{\varepsilon }}^{2}=.03$

*V*, Cramer's *V*; ${\hat{\varepsilon }}^{2}$, Epsilon.

In the group “continuously organized sport”, more than half of the parents were highly educated (mothers with a high educational level: 64.2%, fathers with a high educational level: 57.3%) while the higher educated parents in “no organized sport” showed the lowest percentage across all groups (mothers with a high educational level: 35.0%, fathers with a high educational level: 26.3%). With regard to the FHC_PA_, differences were found between the change patterns [*F*_(3, 493)_ = 5.63, *p* = .001]. The highest FHC_PA_ values were found in the group “continuously organized sport” (*M* = 1.85, SD = .45) and lowest FHC_PA_ values in the group “no organized sport” (*M* = 1.63, SD = .52) (Table 3).

### 3.2 Prediction of PA changes

Results of multinomial logistic regression for changes of organized and overall PA are shown in Tables 4, 5.

**Table 4:** Multinomial logistic regression for change patterns of overall PA.

Predictors	Change patterns								
	Increase^a^			Decline^a^			Continuously insufficient PA^a^		
	Coefficient	*p*	Odds ratio	Coefficient	*p*	Odds ratio	Coefficient	*p*	Odds ratio
Sex*	.508	.119	1.662	.630	.051	1.877	.748	.002*	2.113
Maternal education level*	.461	.146	1.585	.022	.922	1.022	.522	.034*	1.685
Paternal education level	.439	.121	1.551	.010	.973	1.010	.097	.668	1.102
FHC_PA_	−.392	.237	.676	−.270	.411	.763	−.460	.089	.631

^a^ Reference category: continuously sufficient PA.

* Significant at the level of .05.

**Table 5:** Multinomial logistic regression for change patterns of organized sports.

Predictors	Change patterns								
	Change from no organized to organized sport^a^			Change from organized to no organized sport^a^			No organized sport^a^		
	Coefficient	*p*	Odds ratio	Coefficient	*p*	Odds ratio	Coefficient	*p*	Odds ratio
Sex	.442	.077	1.556	.576	.099	1.779	.462	.075	1.588
Maternal education level*	−.147	.558	.863	−.519	.076	.595	−.701	.004*	.496
Paternal education level*	−.320	.131	.726	−.148	.563	.862	−.518	.022*	.596
FHC_PA_*	−.284	.307	.753	−.842	.013*	.431	−.855	.003*	.425

^a^ reference category: continuously organized sport.

* Significant at the level of .05.

Results for change patterns of overall PA showed a significant overall model $\left(\chi^{2}(12)=27.311,p=.007,\mathrm{Nagelkerk}e^{\prime }s\;R^{2}=.059\right)$. Sex and maternal educational level were related to group membership. In relation to the group “continuously sufficient PA” being a girl (*p* = .002, OR = 2.11) and having a highly educated mother (*p* = .034, OR = 1.69) increases the risk of being continuously physically inactive during the course of primary school.

Parents’ educational level and FHC_PA_ significantly predicted group membership of organized PA change patterns (overall model: $\chi^{2}(12)=50.770,\;\;p<\;.001$, Nagelkerke's *R*^2^ = .108). In relation to the group “continuously organized sports” parents’ education and FHC_PA_ were related being in the group “no organized sports”: children with higher educated mothers (*p* = .004, OR = .50), higher educated fathers (*p* = .002, OR = .60) and with a higher FHC_PA_ score (*p* = .003, OR = .43) in grade 1 had a reduced risk of being in the group with no organized sport. In addition, a higher FHC_PA_ at grade 1 (*p* = .013, OR = .43) reduced the risk of changing engagement in organized sports from engaging in organized sports in grade 1 to being not engaged in organized sport in grade 4.

## 4 Discussion

The purpose of this study was to investigate characteristics of four different patterns of PA change from 1st to 4th grade during primary school. The relevance of children's sex, parents' educational level and PA related Family Health Climate (FHC_PA_) as predictors of the changes in overall PA and in engagement in organized sports have been examined. We found differences between overall PA patterns related to parents' educational level and children's sex and between organized sport patterns related to parents' educational level and families FHC_PA_.

Previous research showed a decline of PA with increasing age which already begins during primary school time and starts from around the age of school entry (43). However, PA behavior does not change uniformly across individuals, instead there is an increasing number of studies finding that sub-groups with different change patterns exist (e.g., 11, 44). Existing studies on youth mostly focus on the transition from childhood to adolescence (11, 13, 45, 46) or on transition from adolescence to (young) adulthood (47, 48). There is one study that examines the trajectories from preschool to school years (49). However, to the best of our knowledge, there is no study focusing on the time frame from school entry up to the end of primary school.

Our study compares four possible trajectories over the course of primary school (change from 1st to 4th grade) for two different PA variables (overall PA and engagement in organized sports): no/not enough engagement in organized sports or overall PA at both time points, a change from no/not enough engagement to engagement, a change from (enough) engagement to no/not enough engagement and (enough) engagement at both time points. In our study more than half of the children were—compared to current WHO guidelines—insufficiently physically active at the beginning as well as at the end of primary school. Less than 20% were sufficiently physically active at both time points. This high number of inactive children is consistent with current studies such as the PA Report Cards (50, 51). However, having the longitudinal character as well as the relatively young age (6/7–9/10 years) in mind, this result is even more alarming. This is intensified by the finding, that especially inactive patterns are stable over time (11).

The results for engagement in organized sports differ from the results from overall PA. More than half of the children were engaged in organized sports (e.g., sports club) at both the beginning and the end of primary school. Less than 20% were neither engaged in organized sports in grade 1 nor in grade 4. This is a bright spot as involvement in organized sports can be beneficial for youth development and enhance psychological and social health (52, 53). However, our results indicate that engagement in organized sports seems not to be sufficient for many children to reach the recommended overall PA levels which are associated with physical health benefits. This is in line with a study from Koorts et al. (54) that also showed that sports participation had a small contribution to accelerometer measured MVPA in adolescents.

### 4.1 Change patterns

We used predetermined two-timepoint change patterns based on parent reported overall PA and engagement in organized sports in our study. Other studies that differentiated between different PA trajectories mostly used objectively measured PA and used different types of finite mixture modeling methods (46–49, 55–57). Predominantly, they found between 3 and 5 different trajectories (11). Most studies focused on overall PA or on MVPA, some differentiated between total PA and MVPA (e.g., 14, 49). Besides our study, there is only one study (56, 58) that addressed sports participation along with a measure of PA (here accelerometer measured MVPA). Overall, few studies addressed organized sports participation and found three patterns of change from childhood to adolescence (26, 56, 58–60). The proportion of children in the “no sport participation” group (13.6%) and the “consistent sports participation” group (46.2%) (61) corresponds to our findings (16.1% and 56.1% respectively). Though, the proportion of children in the “dropout from sports participation” group was significantly larger (40.2% vs. 8.2%) which might be due to the older age at the end point (19 years). Howie and colleagues (60) (5 through 17 years) and Findlay and colleagues (26) (4 through 17 years) also found three sub-groups of organized sport trajectories. While Kwon and colleagues (56) and Findlay and colleagues (26) did not find a sub-group that changed from no sports participation to sports participation, Howie and colleagues (60) found this group only for boys with less than 10% of the boys being in this group. However, in our study nearly 20% of the children were in this group (not engaged in organized sports in grade 1 but engaged in grade 4). This difference might at least partially be due to the younger age of our sample. However, it suggests that primary school years might be a good point in time to promote engagement in organized sports. In contrast, the likelihood of joining organized sports for the first time during adolescence seems to be low.

Various studies and systematic reviews have shown that PA decreases during the transition from childhood to adolescence (e.g., 43, 44). Pate and colleagues (14) found that decreases between 10 and 13 are steeper than between 14 and 17 years. Kwon and colleagues (61) found that low levels of PA at age 10 were related to low levels through adolescence and young adulthood. Therefore, the proportion of children in our sample that do not reach the recommendations at grade 4, is likely to increase with increasing age. Children that are in the decline and continuously insufficient group unlikely will increase their PA and reach the recommended PA levels. This suggests that primary school age might be an important time point for implementing interventions.

### 4.2 Predictors of change patterns

Our study found that child's sex, parents' educational level and families FHC differentiates between change patterns. Various factors have been examined in previous studies. Associations to changes in PA from childhood to adolescence have been found for SES, parental education, children's sex, parental support and parents' PA (11, 14, 49).

A large amount of research found sex differences and suggests that girls are less active than boys (6, 62). Accordingly, in our study more boys (62.5%) were in the group “continuously sufficient active” while more girls were in the group “continuously insufficient active” (56.4%). The differences in sports participation were not that pronounced. However, more girls than boys were in the groups that dropped out between first and fourth grade (61%) and in the group that were not engaged in organized sports at the beginning and the end of primary school (57.5%). Studies examining PA trajectory sub-groups showed similar differences (11, 46, 49) and studies focusing on sports participation found equal results as well (26, 60). Howie and colleagues (60) and also Findlay and colleagues (26) found sex-specific trajectories, a “non participator” group was found only for girls with 18.1% of the girls being in this group which corresponds to our results (17.9%). Kwon and colleagues (56) did not find differences in the sex distributions of their sports participation trajectories.

The “gender gap” in PA is a persistent issue for many years (63, 64) and the reasons for the differences to the detriment of girls are still poorly understood (65). There is a need for research aiming to identify sex-specific barriers and facilitators of different domains and types of PA from early childhood through to adulthood. Although sex differences are well documented, interventions often do not consider the factor sex sufficiently (66, 67).

Besides sex differences, disparities in PA engagement with regard to socioeconomic background have often been found (68). However, results of single studies are inconsistent, especially for children and adolescents (20, 22) and these inconsistencies might be due to differences related to PA domains (69). For overall PA we found an association of mothers' (but not fathers) educational level in the counterintuitive direction that a high educational level is associated with being in the group that does not achieve recommended overall PA levels at the beginning and the end of primary school. This result is in line with Pate and colleagues (14) who found that children of less educated parents were more active than children of high educated parents. There are further studies indicating that high educational levels have an inverse relation to children's PA (18, 20). Explanatory approaches suppose that highly educated parents might encourage or put pressure on children and adolescents to engage in academic tasks which might be associated with a higher workload related to school tasks (e.g., longer school days, more demanding homework) and thus less free time available for PA (69). Furthermore, higher educated parents might prioritize children's organized sports which could be associated with less priority of engaging in unstructured PA such as outdoor play (18, 70). In line with this, we found for sports participation an inverse association, higher educational levels of both parents are related to engaging in organized sports over the course of primary school. This is also consistent with the findings of systematic reviews and meta-analyses, which suggest that socioeconomic disparities are greater and more pronounced in sport than in total PA (20, 69).

Previous research highlighted the role of the family environment as a key source of influence on children's and adolescents' PA (21, 71). Trajectory studies previously examined some family factors and found associations with parental support and parent PA (e.g., 13, 49, 58, 72). A recent study found that parental encouragement and support are important influences on children's PA during the transition from childhood to adolescence (13). However, we are not aware of studies that included other family environmental factors than parental factors. This is surprising and points to a research gap. The family is the basic social context where behavioral patterns develop and are maintained (21). Research in the field of health-related behaviors such as PA mostly focuses on parents' influence on children's PA, and studies demonstrated the gate-keeping role of parents (73). However, there are family-level socialization dynamics beyond parent-child influences. There are some family-level constructs such as FHC and “family functioning” that have been examined in a small number of studies. In line with previous studies utilizing the FHC we found that the FHC predicts group membership in the change patterns of organized sports. A higher FHC was related to an increased likelihood of continuous sports participation. However, there was no association with overall PA change patterns. Nevertheless, this is quite promising and future studies should consider family-level constructs as relevant predictors of PA trajectories. Besides these family-level constructs, further parent-child factors should be included as well, for example parenting styles and parenting practices (28).

The findings of our study—as well as previous research—that the associations between sex, parents' educational level, FHC_PA_ and change patterns differed depending on overall PA and sport participation, points to the relevance of differentiating PA types and domains. A systematic review of Kemp and colleagues (74) showed that longitudinal changes differ for organized PA, non-organized PA, active transport and active chores. However, most studies examining PA trajectories focused on overall PA and/or MVPA (11), studies addressing VPA [e.g., (75)] or different domains of PA are rare.

### 4.3 Limitations and future directions

The current study has some limitations that have to be mentioned. Firstly, we used subjective measures for overall PA and organized sports participation. While for the latter there isn't any option to assess this domain objectively, for overall PA there are objective alternatives. Assessing children's PA via proxy report by parents has weaknesses such as recall bias and social desirability. Objective measurements provide accurate information regarding the types/intensities of PA. However, these measures are limited to a short time frame while self-report measures can better address habitual PA (76). Furthermore, accelerometer data does not allow for a differentiation between PA domains. Therefore, further studies examining PA changes could benefit from a combination of accelerometer measured and self-reported PA. Secondly, we used only two time points and *a priori* defined patterns for describing changes over time. The correspondence of our results with that of trajectory studies using multiple measurement points and applying finite mixture modeling methods (e.g., latent class analysis, latent class growth analyses, growth mixture modeling and group-based trajectory modeling) indicates that this approach is appropriate. However, future studies should consider several measurements points over the course of primary school, for example one measurement per year from grade 1 to grade 4. Thirdly, the sample was better educated than the average German population (77) possibly limiting the generalizability of the findings. Therefore, replicating the results in other samples or cultures is desirable. Lastly, in this study, parental education was used as an indicator of socio-economic status (SES). While a more comprehensive SES index—including income and occupational status—would have been more informative, education is the most commonly used and internationally comparable SES proxy and suitability for cross-study comparisons.

However, our study has several strengths that are worth emphasizing. Firstly, the focus on primary school age: Previous studies mostly focused on the transition of childhood to adolescence or adolescence to (young) adulthood. Studies focusing on changes in childhood are rare and studies focusing on the first years in the educational system, representing a time of massive changes and dynamic development, are lacking. Secondly, the consideration of two different types of PA (overall PA and organized sports participation): There are only a few trajectory studies that included more than one type of PA. These studies found different patterns depending on the type of PA. Thirdly, the inclusion of a family-level factor: Family-level variables such as the FHC_PA_ seems to be relevant for shaping changes of PA. Previous studies focused on parental support and parents' PA.

### 4.4 Implications

Along with other studies (e.g., 11, 13, 49, 78) our findings indicate that programs should start in early years. The school entry which is accompanied by a development boost might be a time point of particular importance (14). Furthermore, interventions should consider sex differences and should especially aim to promote girls' PA (65, 67). In addition, interventions have to consider the relevance of the family for children's PA changes. At best, interventions address these influences by including families/parents into interventions. However, research has shown how challenging this is (79). Next, interventions should consider that different types and domains of PA might need different approaches as they change differently over time and differ depending on sex and socioeconomic background factors such as educational level. For example, increasing the perceived relevance of unstructured PA in parents might be adequate especially for children with highly educated parents and facilitating the access to organized sports might be an approach for children from low SES families.

## 5 Conclusion

Along with several trajectory studies, we found that a child's sex and parents' educational level predicted membership in change patterns of overall PA and engagement in organized sports from beginning to end of primary school. Additionally, our study found evidence for the relevance of a family-level variable—Family Health Climate related to PA (FHC_PA_)—that predicted changes in organized sports participation. This is the first study focusing on change patterns during primary school and it complements previous findings of an early decline of PA. The entry in the educational system might be a good time to start with interventions. The study highlights the importance of differentiating between different types and PA domains as sex, educational level and FHC_PA_ differently predicted membership in change groups for overall PA and sports participation. The findings comprise valuable information for the development and implementation of intervention programs. Interventions should start at early years, differentiate types and domains of PA and address them differently.

## Data Availability

The dataset presented in this study is available upon reasonable request from the Faculty of Sport Science, Leipzig University, Leipzig, Germany. Contact: petra.wagner@uni-leipzig.de.
